# Humoral Response to the *Anopheles gambiae* Salivary Protein gSG6: A Serological Indicator of Exposure to Afrotropical Malaria Vectors

**DOI:** 10.1371/journal.pone.0017980

**Published:** 2011-03-17

**Authors:** Cinzia Rizzo, Raffaele Ronca, Gabriella Fiorentino, Federica Verra, Valentina Mangano, Anne Poinsignon, Sodiomon Bienvenu Sirima, Issa Nèbiè, Fabrizio Lombardo, Franck Remoue, Mario Coluzzi, Vincenzo Petrarca, David Modiano, Bruno Arcà

**Affiliations:** 1 Department of Public Health and Infectious Diseases, “Sapienza” University, Rome, Italy; 2 Istituto Pasteur - Fondazione Cenci Bolognetti, Sapienza University, Rome, Italy; 3 Department of Structural and Functional Biology, “Federico II” University, Naples, Italy; 4 UR016 Biology and Control of Vectors, Institut de Recherche pour le Développement, Montpellier, France; 5 Centre National de Recherche et de Formation sur le Paludisme, Ouagadougou, Burkina Faso; 6 Department of Biology and Biotechnology “Charles Darwin”, Sapienza University, Rome, Italy; Lile 2 University, France

## Abstract

Salivary proteins injected by blood feeding arthropods into their hosts evoke a saliva-specific humoral response which can be useful to evaluate exposure to bites of disease vectors. However, saliva of hematophagous arthropods is a complex cocktail of bioactive factors and its use in immunoassays can be misleading because of potential cross-reactivity to other antigens. Toward the development of a serological marker of exposure to Afrotropical malaria vectors we expressed the *Anopheles gambiae* gSG6, a small anopheline-specific salivary protein, and we measured the anti-gSG6 IgG response in individuals from a malaria hyperendemic area of Burkina Faso, West Africa. The gSG6 protein was immunogenic and anti-gSG6 IgG levels and/or prevalence increased in exposed individuals during the malaria transmission/rainy season. Moreover, this response dropped during the intervening low transmission/dry season, suggesting it is sensitive enough to detect variation in vector density. Members of the Fulani ethnic group showed higher anti-gSG6 IgG response as compared to Mossi, a result consistent with the stronger immune reactivity reported in this group. Remarkably, anti-gSG6 IgG levels among responders were high in children and gradually declined with age. This unusual pattern, opposite to the one observed with *Plasmodium* antigens, is compatible with a progressive desensitization to mosquito saliva and may be linked to the continued exposure to bites of anopheline mosquitoes. Overall, the humoral anti-gSG6 IgG response appears a reliable serological indicator of exposure to bites of the main African malaria vectors (*An. gambiae*, *Anopheles arabiensis* and, possibly, *Anopheles funestus*) and it may be exploited for malaria epidemiological studies, development of risk maps and evaluation of anti-vector measures. In addition, the gSG6 protein may represent a powerful model system to get a deeper understanding of molecular and cellular mechanisms underlying the immune tolerance and progressive desensitization to insect salivary allergens.

## Introduction

It is well known that blood sucking arthropods salivate injecting into their hosts, while feeding, a complex cocktail of bioactive factors of essential importance for the successful acquisition of the blood meal [1]. The main role of these components is to antagonize the physiological responses of the host to tissue injury, namely hemostasis, inflammation and immunity [2], [3]. However, independently from their biochemical properties, these factors also elicit into the host a humoral response and, hence, circulating anti-salivary proteins antibodies can be detected in the sera of individuals repeatedly bitten by arthropods. This anti-saliva antibody response may represent a useful tool to evaluate host exposure to arthropod vectors of diseases such as ticks [4], sand flies [5], triatomines [6], tsetse flies [7], [8] and mosquitoes [9]–[13].

So far, large-scale epidemiological analyses based on the anti-saliva immune response, have been hindered by the difficulty of obtaining large amounts of saliva or salivary extracts (which is tedious, difficult to standardize and poorly reproducible) and, more importantly, by the complex nature of blood sucking arthropod saliva, that may give rise to potentially misleading cross-reactivity to other antigens. Transcriptome and proteome analyses in the last 5–10 years highlighted that the salivary repertoires of blood-feeding insects consist both of proteins that are widely shared between insect families and of proteins that are family-, genus- and even species-specific [3], most probably as result of the convergent evolutionary nature of hematophagy and of the unusually fast evolutionary rate of salivary proteins [14]–[16]. Comparative analysis of sialotranscriptomes from different *Culicidae* family members allowed for the identification of a large group of *Anopheles*-specific proteins, i.e. not found in *Culex* or *Aedes* mosquitoes, and viceversa [17]–[19]. These proteins, if immunogenic, may represent ideal candidates for the development of serological markers of exposure to anopheline mosquitoes.

We focused the attention on one of these *Anopheles*-specific proteins, which we previously identified and named as gambiae Salivary Gland protein 6 (gSG6, GI: 13537666) [20]. This is a small polypeptide (∼10 kDa) specifically expressed in adult female salivary glands, relatively abundant in saliva and with no significant similarity to any other known protein. The precise function of gSG6 is still undetermined, although its depletion from mosquito saliva by RNAi affects probing time and blood feeding ability [21]. In this study we report the purification of the *Escherichia coli-*expressed gSG6 and its use for a large scale epidemiological analysis on sera of individuals from a malaria hyperendemic area of Burkina Faso, West Africa.

## Results

**Figure 1:**
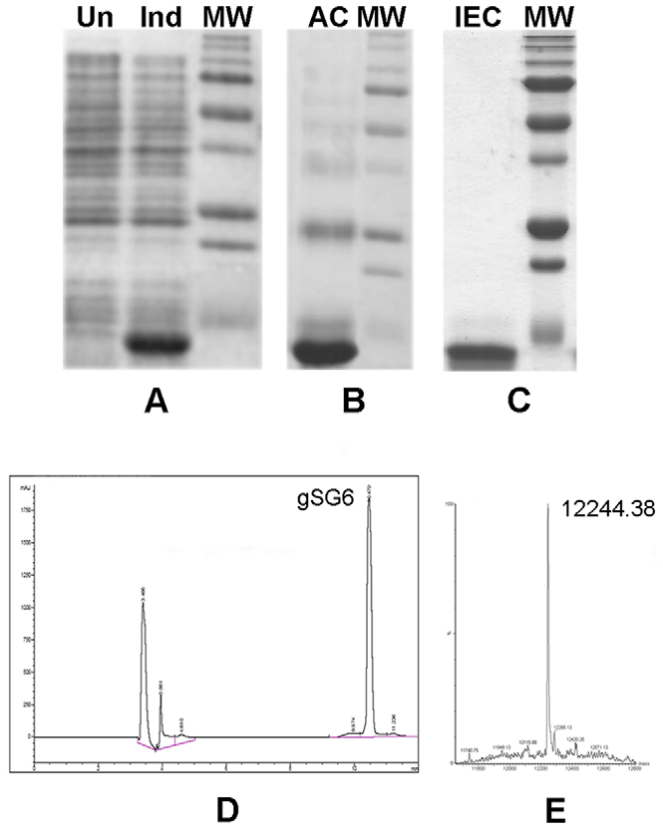
Expression and purification of the *An. gambiae* gSG6 salivary protein. Recombinant gSG6 was expressed in *E. coli* Bl21(DE3)RIL cells. **(A)** SDS-PAGE analysis of protein fractions from uninduced (Un) and induced cells (Ind). **(B)** SDS-PAGE analysis of pooled protein fractions obtained after His-Trap affinity chromatography (AC) under denaturing conditions. **(C)** Fractions containing purified gSG6 after anion exchange chromatography (IEC) were pooled and analyzed by SDS-PAGE. **(D)** Homogeneity of the purified protein was confirmed by Reverse Phase HPLC. The first peak is artifactual and the second one corresponds to the gSG6 recombinant protein. **(E)** Mass Spectrometry, the mass corresponding to the recombinant gSG6 is indicated. MW, Molecular Weight Markers.

A *Pichia pastoris*-expressed version of the *An. gambiae* gSG6 [21] was previously used to measure the anti-gSG6 IgG response in a small group of sixteen Senegalese children living in a malaria endemic area. This initial test provided encouraging preliminary information on the immunogenicity of the protein [22]; however, the *Pichia*-expressed gSG6 generated a relatively high background in control sera (i.e. sera from individuals not exposed to *Anopheles* bites) even though it was apparently pure and not glycosilated [21] (B.A., unpublished observations). Nevertheless, the promising results obtained with a gSG6-based peptide [22], [23] prompted us to express the protein in *E. coli*. The recombinant protein has a predicted molecular mass of ∼12.4 kDa (108 aa) and carries an additional 21 aminoacids at the N-terminus (His-tag and thrombin site). Purification was achieved by His-Trap affinity chromatography in denaturing conditions, followed by refolding of the protein and by anion-exchange chromatography (Figure 1, panels A–C). The purity and correct refolding of the recombinant gSG6 was confirmed by reverse-phase high-pressure liquid chromatography (RP-HPLC) and mass spectrometry (MS) (Figure 1, panels D–E). MS analysis yielded a major peak corresponding to a mass of 12244.4, which fits very well with the mass expected for the recombinant gSG6 lacking the starting Met and with the ten Cys residues involved in disulfide bridges (12243.6).

**Figure 2:**
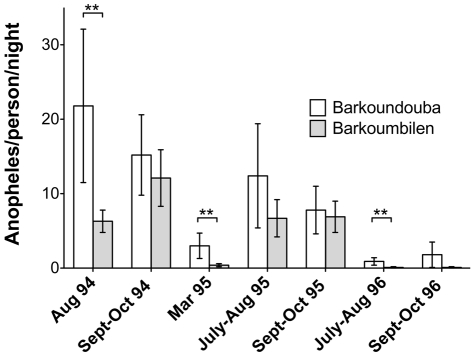
Anopheles density in the villages of Barkoundouba and Barkoumbilen. The density is expressed as the mean of anopheline mosquitoes (*An. gambiae sensu lato* + *An. funestus*) per person per night. Whiskers denote the 95% Confidence Interval (CI). The number of catches (n) in Barkoundouba and Barkoumbilen in the different surveys were, respectively: August ’94 (n = 12, n = 12), September-October ’94 (n = 24, n = 24), March ’95 (n = 12, n = 12), July-August ’95 (n = 26, n = 28), September-October ’95 (n = 36, n = 36), July-August ’96 (n = 26, n = 28), September-October ’95 (n = 23, n = 22). P value was determined according to Student's two-tailed t-test (**, 0.001<p<0.01).

Anti-gSG6 IgG were measured by Enzyme-Linked ImmunoSorbent Assay (ELISA) in 1752 human sera collected in two villages of a rural malaria hyperendemic area of Burkina Faso during three consecutive years, at the beginning and at the end of each high transmission rainy season (August and October ‘94, ‘95 and ‘96), and during one of the intervening low transmission dry season (March ‘95). Sera from 42 Europeans non-exposed to bites of anopheline mosquitoes were used as controls. It is worth pointing out two aspects with relevant epidemiological implications. First, the two villages under study are inhabited by ethnic groups with different immune reactivity and susceptibility to *Plasmodium falciparum* malaria [24]: the Fulani living in Barkoundouba and the Mossi in Barkoumbilen. Second, insecticide-treated curtains (ITC) were distributed and applied in the two villages just before the 1996 high transmission season (June) [25]. The villages were under entomological monitoring during the study periods and, as previously reported, *P. falciparum* inoculation rates were comparable in Barkoundouba and Barkoumbilen [25]. The number of *Anopheles*/person/night, as measured by the indoor pyrethrum spray catch method, appeared higher in Barkoundouba as compared to Barkoumbilen; however, only in August ’94, March ’95 and July-August ’96 this difference was statistically significant (Figure 2). The decreased vector density observed in July-October ‘95, in comparison to August-October ‘94, was most likely a consequence of the lower rainfall [24]. Overall a clear drop in *Anopheles* density was evident both during the dry season (March ’95) and after application of ITC (July-August ’96, September-October ’96).

### gSG6 immunogenicity

**Figure 3:**
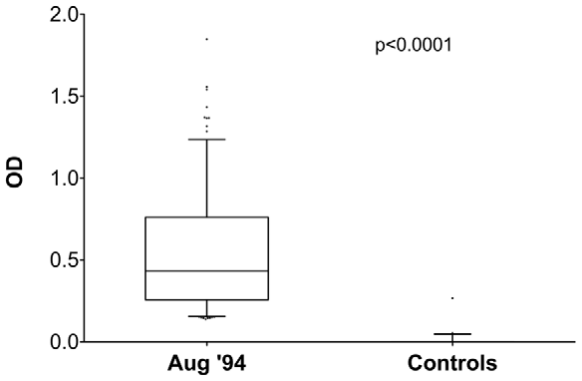
Anti-gSG6 IgG levels in exposed individuals and in non-exposed controls. Box plot of OD values in European non exposed controls and in responders from the August ’94 survey. Number of individuals included in the analysis (n), average age in years ±95% CI were as follows: Europeans, n = 42 (33.4±6.1); August ‘94, n = 197 (15.0±2.0). Box plots display the median OD value, 25^th^ and 75^th^ percentile. Whiskers represent 5–95 percentile and dots the outliers. P value was determined according to Mann-Whitney test.

Anti-gSG6 IgG prevalences and levels were significantly higher in the sera of individuals exposed to *Anopheles* bites (Burkinabé) as compared to non-exposed controls (European). As an example the OD values among responders (i.e. individuals with OD values above the cut-off) from the August ‘94 survey and in control subjects are reported in Figure 3. Very similar patterns and identical highly statistical differences (p<0.0001) were also found when the remaining six surveys and/or all the individuals, rather than just the responders, were analyzed (not shown). These observations show that the *An. gambiae* gSG6 is immunogenic, evokes in exposed individuals an IgG antibody response and, importantly, that no appreciable cross-reactivity is detectable in sera of individuals non-exposed to bites of anopheline mosquitoes.

### Seasonal variation of the anti-gSG6 IgG response

**Figure 4:**
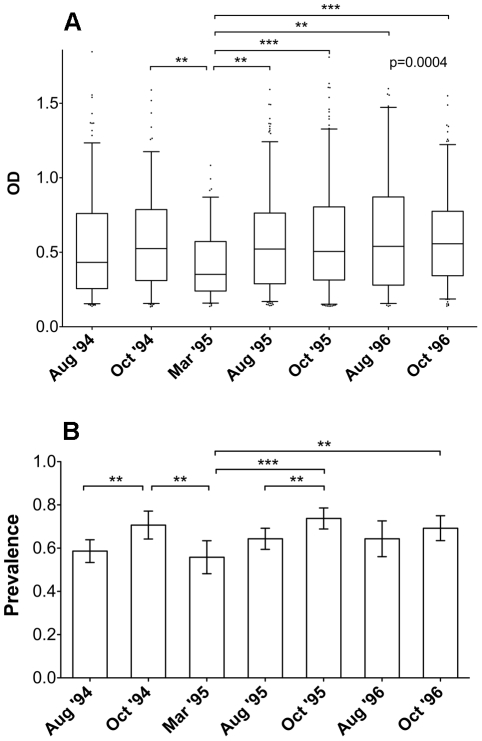
Seasonal variation of the anti-gSG6 IgG response. **(A)** Box plot of OD values in the responders from the different surveys. Number of individuals, average age ±95% CI, box plots and whiskers as in Figure 3. August ’94, n = 197 (15.0±2.0); October ’94, n = 135 (14.8±2.5); March ’95, n = 91 (15.0±3.0); August ’95, n = 238 (14.2±1.8); October ’95, n = 233 (15.1±1.9); August ’96, n = 83 (13.1±2.4); October ’96, n = 171 (16.4±2.3). P value was determined according to Kruskal-Wallis test. Pairwise comparisons refer to Dunn's multiple comparison test (**, 0.001<p<0.01; ***, p<0.001). **(B)** Seasonal variation of seroprevalence of anti-gSG6 IgG. The frequency of responders during the seven different surveys is reported. Whiskers denote the 95% CI. August ’94, n = 336 (19.0±1.9); October ’94, n = 191 (16.2±2.3); March ’95, n = 163 (17.1±2.4); August ’95, n = 370 (15.8±1.5); October ’95, n = 316 (16.5±1.7); August ’96, n = 129 (18.2±2.8); October ’96, n = 247 (19.3±2.3). P values were determined by Yates-corrected chi square test (**, 0.001<p<0.01; ***, p<0.001).

When the anti-gSG6 IgG response was compared in the seven different surveys a seasonal variation was readily observed. As a general pattern the anti-gSG6 IgG response increased, or persisted at a high stationary level, during the transmission/rainy season (August to October), when there is a continued exposure to bites of anopheline mosquitoes. This trend appeared already evident by plotting the OD levels of responders in the different surveys (Figure 4A), and it was even more pronounced by looking at the prevalence (Figure 4B). The frequency of responders increased significantly from the beginning (August) to the end (October) of the transmission season both in the 1994 and 1995 surveys (p≤0.01), whereas no significant increase was found during 1996, perhaps because of the lower indoor mosquito density due to the ITC applied in June ‘96. Remarkably, both anti-gSG6 antibody levels and prevalence dropped significantly during the dry season (March ‘95) to increase again during the following transmission/rainy periods (August-October ’95), and these changes were highly significant (Figure 4, p<0.01). Overall, the pattern of seasonal variation parallels the exposure to anopheline vectors and malaria transmission. Moreover, the drop during the intervening dry season implies that the anti-gSG6 IgG response is not long-lasting and tends to decrease after a few months of lower or absent exposure.

### Anti-gSG6 IgG response in the Mossi and Fulani ethnic groups

**Figure 5:**
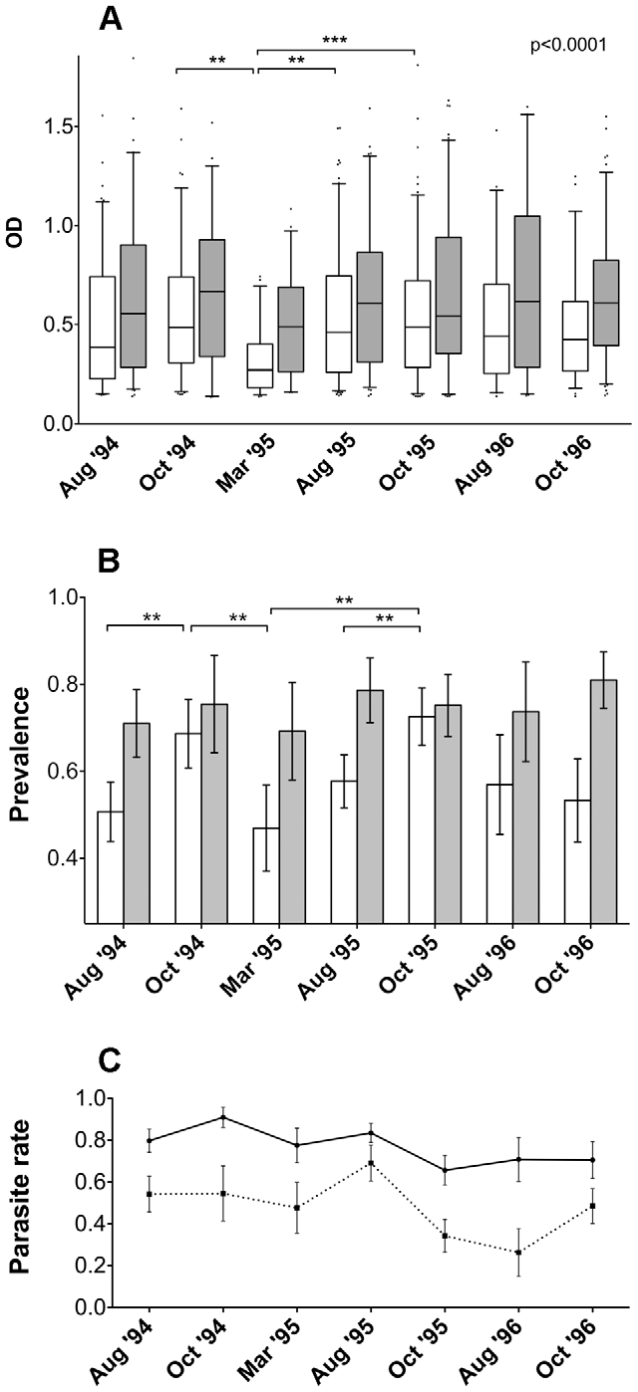
Anti-gSG6 humoral response in the ethnic groups Mossi and Fulani. **(A)** Box plot of OD values among responders in the seven different surveys (Mossi, empty boxes; Fulani, grey boxes). The number of Mossi (M), Fulani (F) and the average age ±95% CI were as follows: August ’94 M = 104 (16.1±3.1), F = 93 (13.8±2.6); October ’94 M = 92 (13.4±2.8), F = 43 (17.7±5.2); March ’95 M = 46 (18.7±4.9), F = 45 (11.2±3.1); August ’95 M = 146 (16.1±2.8), F = 92 (11.0±1.6); October ’95 M = 127 (12.8±2.2), F = 106 (17.8±3.1); August ’96 M = 41 (13.6±3.6), F = 42 (12.7±3.2); October ’96 M = 56 (14.5±3.2), F = 115 (17.3±3.1). Box plots, whiskers and p values as in Figure 4A. **(B)** Prevalence of antibodies to gSG6 in Mossi (empty columns) and Fulani (grey columns) in the seven different surveys. Whiskers and p values as in Figure 4B. The number of Mossi (M), Fulani (F) and the average age ±95% CI were as follows: August ’94 M = 205 (19.8±2.6), F = 131 (17.7±2.9); October ’94 M = 134 (14.0±2.4), F = 57 (21.5±5.0); March ’95 M = 98 (17.4±3.1), F = 65 (16.7±3.7); August ’95 M = 253 (17.5±2.1), F = 117 (12.0±1.5); October ’95 M = 175 (13.7±2.0), F = 141 (19.9±2.9); August ’96 M = 72 (18.4±3.8), F = 57 (17.9±4.1); October ’96 M = 105 (18.3±3.3), F = 142 (20.0±3.1). **(C)** *Plasmodium* parasite rates in Mossi (solid line) and Fulani (dotted line) in the seven different surveys. More than 95% of infections were due to *P. falciparum*. Bars denote the 95% CI. Numbers of individuals and average age as in B.

When the data shown in Figure 4 were plotted stratifying for the two different ethnic groups the Fulani revealed a constantly higher anti-gSG6 IgG response (Figure 5A), and this difference was statistically significant in most surveys (August ’94, March ’95, October ’95 and October ’96; Mann-Withney, 0.0408>p>2e-4). Similarly, the Fulani always exhibited a higher prevalence (Figure 5B), which also was significant in most cases (August ’94, March ’95, August ’95 and October ’96; Yates’ chi-square, 0.008>p>6e-6). This higher anti-gSG6 humoral response was not unexpected because, as previously reported, Fulani display a higher immune reactivity as compared to the sympatric ethnic groups Mossi and Rimaibé and are more resistant to malaria infection [24]. A detailed account of the parasitological status of the population under study has been described elsewhere [24], [25], however, we shortly summarize here *Plasmodium* parasite rates in the Mossi and Fulani ethnic groups during the seven surveys (Figure 5C). Remarkably, the seasonal variation of the anti-gSG6 IgG response was less pronounced in the Fulani, although the trend appeared essentially the same in both ethnic groups. Comparison of both anti-gSG6 IgG levels and prevalence between the dry period (March ’95) and the previous or following transmission/rainy seasons yielded a significant difference only in the Mossi, never in the Fulani (Figure 5A and 5B). Finally, it is worth noting that the prevalence significantly increased in the Mossi from the beginning to the end of the transmission/rainy season both in the 1994 and in the 1995 surveys (Yates' chi-square, p = 0.0016 and p = 0.0023 respectively); on the contrary, no difference in the prevalence was observed between August (0.57) and October (0.53) following the set-up of ITC in June ‘96 (Figure 5B). This different trend appears fully compatible with a reduced indoor vector density and the consequential expected reduced exposure to *Anopheles* bites.

### Pattern of the anti-gSG6 IgG response according to age

**Figure 6:**
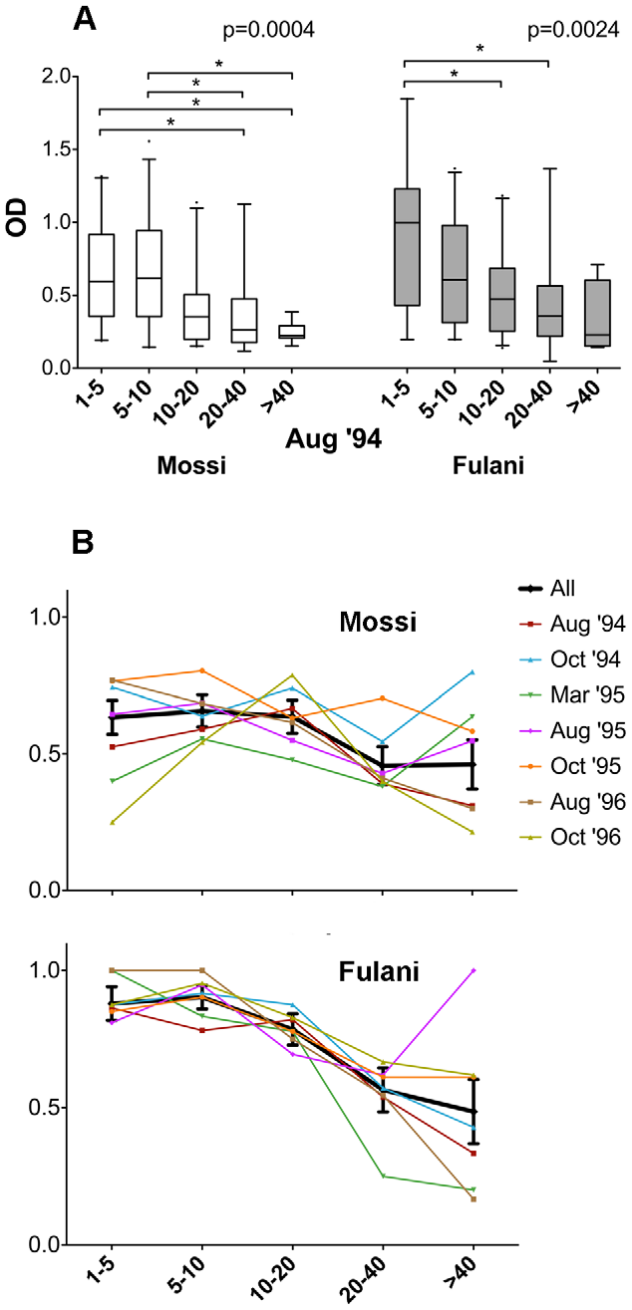
Anti-gSG6 IgG response by age in the ethnic groups Mossi and Fulani. **(A)** Box plot of OD values among responders in the August ’94 survey (Mossi, empty boxes; Fulani, grey boxes). Age groups are indicated at the bottom (Mossi: 1–5, n = 20; 5–10, n = 26; 10–20, n = 32; 20–40, n = 18; >40, n = 9. Fulani: 1–5, n = 19; 5–10, n = 25; 10–20, n = 32; 20–40, n = 14; >40, n = 4). Box plots, whiskers and p values as in Figure 4A (*, 0.01<p<0.05). **(B)** Prevalence of antibodies to gSG6 in Mossi (top) and Fulani (bottom) in the seven different surveys according to age (thin coloured lines). A cumulative trend was obtained pooling together the data of all the surveys (black tick line with 95% CI). The number of individuals analyzed for each survey are included as supplementary information (Text S1).

It is widely known that in endemic areas the humoral response to *Plasmodium falciparum* antigens develops gradually, being lower in children under five years of age and progressively increasing to reach the highest level in adults. This age-related profile of increased seroprevalence and antibody levels against different *P. falciparum* antigens (CSP, TRAP, MSA-1, Pf155, Pf332) has also been reported for the same individuals analyzed in this study [24], [26]. When the anti-gSG6 IgG response was stratified into five different age groups, both the Mossi and the Fulani exhibited an age-dependent decrease, rather than increase, in the humoral response. This was readily evident for the August ‘94 sample when the anti-gSG6 IgG levels were plotted according to the different age-groups (Figure 6A) and similar patterns were observed for the other surveys (not shown). Actually, in all cases the median OD values among the responders was higher in children (1–10 years) than in adults (>20 years) and in most cases (8/14) the difference was statistically significant (Table S1). A parallel trend was observed when the seroprevalence was analyzed, as shown by the independent analysis of the seven different surveys, both in the Mossi and in the Fulani (Figure 6B, coloured thin line). The frequency of responders was higher in children aged 1–10 years than in adults (>20 years old) in 13 out of 14 cases and the difference was in most cases statistically significant (10/14, Table S1). A cumulative trend, which may help overcoming possible bias due to the small size of some samples, was obtained pooling together the data of all the surveys, (Figure 6B, black tick line). Overall these observations clearly show that the anti-gSG6 IgG response decreased in exposed individuals after approximately ten years of age; this decrease was more evident in the Fulani, who exhibit higher antibody levels and higher seroprevalence, especially in young children.

## Discussion

With almost 250 million cases and roughly a million deaths among children (WHO 2008, http://www.who.int/malaria/en/) malaria is still a devastating disease, especially in sub-Saharan Africa where *P. falciparum* and *An. gambiae*, the most deadly parasite-vector combination, are widely spread. The assessment of transmission intensity, the determination of spatial and seasonal variation of mosquito density and the evaluation of both vector and parasite control interventions are of paramount importance for the implementation of effective malaria control programs. The gold standard classical method for the assessment of malaria transmission intensity is the determination of the entomological inoculation rate (EIR), which measures human exposure to parasite-carrying mosquitoes. However, entomological measures are labor-intensive, expensive and may be difficult to apply (i.e. when mosquito numbers are low) or face logistic constraints. Alternative methods to estimate *Anopheles* density would be extremely valuable, allowing for epidemiological studies also when the use of classical entomological methods would be difficult or impossible. Serological methods to estimate malaria transmission intensity based on the antibody response to parasite antigens have been developed or are under current development [27]–[30] and, therefore, the parallel measure of the antibody response to *Anopheles* salivary antigens would be especially convenient [31]. In addition, the development of serological tools would also allow for the assessment of *Anopheles* exposure in children, which is ethically unfeasible by human landing catches, the method traditionally used with adult volunteers. Moreover, the malaria burden is currently undergoing a phase of decline in several areas of sub-Saharan Africa [32], most likely because of the improved prevention, diagnosis and treatment following the increased financial support of the last few years (i.e. enhanced availability of insecticide-treated-bednets and/or of artemisinin combination therapies). The availability of serological markers of exposure to *Anopheles* bites would represent a further complementary tool especially in low malaria transmission areas and for the monitoring of control interventions based on anti-vector measures.

The correlation of anti-saliva antibody response to *Anopheles* density and disease risk has been previously reported [10]–[13], although further evaluation and development of the methodology has been limited by the difficulty of obtaining immunogenic recombinant salivary proteins in large amounts. The availability of large amount of information from the salivary transcriptomes of mosquitoes and other blood feeding arthropods [3], [19] is offering new opportunities for the development of recombinant salivary proteins as epidemiological markers of exposure to disease vectors, as recently shown for the sand fly *Lutzomyia longipalpis*, a vector of the *Leishmania* parasite [33].

We expressed and purified the anopheline-specific gSG6 and we report here the first large scale analysis of the humoral response to a recombinant *An. gambiae* salivary protein. In our surveys the entomological data are based on pyrethrum spray catches, a method that can be less accurate as compared to human landing catches on adult volunteers. However, even within their possible lower degree of accuracy, our measurements clearly and reliably document the sharp decrease in *Anopheles* density both during the dry season (March ‘95) and after application of ITC (July-October ‘96). Our study confirms the immunogenicity of the *An. gambiae* gSG6 and shows the absence of significant cross-reactivity to other widespread antigens. Moreover, the recombinant protein appears at least three to fivefold more sensitive than the gSG6-P1 peptide used in previous studies [22], [23], as indicated both by our comparative tests (not shown) and by the different serum dilutions needed to work with the peptide and with the recombinant protein (1∶20 and 1∶100, respectively).

Both the anti-gSG6 IgG levels and the seroprevalence increased with the progression of the rain-driven malaria transmission (August to October ‘94), a pattern that is fully in agreement with the persistent exposure to *Anopheles* bites. The response dropped during the following dry season (March ‘95), when the mosquito density becomes very low, to increase again during the following transmission/rainy season (August and October ‘95). These observations show that the antibody response to the gSG6 protein exhibits two important features that a potential marker should possess: (i) it is sensitive to the seasonal variation of human exposure to mosquito bites, and (ii) decreases significantly after a few months of reduced or absent exposure, although the maintainance of relatively high anti-gSG6 IgG levels and prevalence was recorded during the dry season in March ‘95. Both the dynamics of the anti-gSG6 IgG response, which may require a longer period to vanish, and the specific individual response may contribute to this maintainance. However, the decrease during the dry season was statistically significant, suggesting that the immune response to the gSG6 protein may be sensitive enough to evaluate variation in vector density. Similar seasonal fluctuations of the antibody response to *An. gambiae* saliva have been recently reported in an epidemiological study in Angola [34]; moreover, a drop in the anti-saliva IgG response has been previously described in a cohort of French soldiers, three months after their return from a travel period in tropical Africa [12]. Therefore, importantly, the overall pattern of the anti-gSG6 IgG response appears to accurately reproduce the humoral response to whole saliva. In addition, this study sets some principles that encourage the search for additional immunogenic *Anopheles*-specific salivary proteins, which may exhibit more suitable and/or desirable properties.

**Figure 7:**
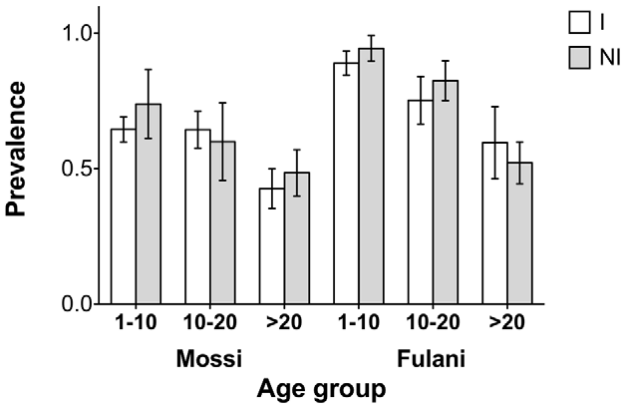
Prevalence of anti-gSG6 IgG response in *Plamodium* infected and non-infected individuals. Data from the seven different surveys were pooled together and analyzed according to ethnic group (Mossi and Fulani), age group (1–10 years, 10–20 years, >20 years) and malaria infection status (I, infected; NI, non-infected).

The higher anti-gSG6 IgG response observed in the Fulani as compared to the Mossi was not unexpected since this ethnic group was previously found to exhibit a more powerful humoral response to *P. falciparum* antigens [24] as well as to other pathogens (D.M., unpublished observations). This feature was more recently suggested to be a consequence of a functional deficit of T regulatory cells [35]. Importantly, individuals of the Mossi ethnic group showed a significant variation of the anti-gSG6 antibody response both during the progression of the transmission/rainy season and in the transitions rainy-dry and dry-rainy periods. On the contrary, Fulani appeared to keep a higher humoral response over time and, for this reason, the seasonal variations of the anti-gSG6 IgG response were less pronounced than in the Mossi and did not reach statistical significance. It is known that malaria infection might negatively affects immune responses and, for this reason, it may be argued that the different malaria status between the two groups, Mossi and Fulani, may be responsible of the differential anti-gSG6 IgG response reported here. However, the contribution of the different malaria status, if any, must be negligible because the prevalence of responders to gSG6 in the different age classes does not show any significant variation between infected and non-infected individuals in both ethnic groups (Figure 7). These observations suggest that the human genetic background should be taken into account to achieve higher sensitivity while evaluating vector exposure through the antibody response to saliva or salivary antigens.

In our study area the application of ITC strongly decreased indoor mosquito density. This reduction appeared to affect the humoral response to gSG6, as indicated by the absence of any significant change in prevalence in the period from August to October ‘96, which is in contrast with the observations concerning the Mossi during the previous 1994 and 1995 rainy/transmission seasons (Figure 5A and 5B). However, the impact on both IgG levels and prevalence is much lower as compared to the dry period in March ’95, when a similar drop in vector density was reported. This difference may depend from the selective effect of ITC on indoor mosquito biting, whereas the outdoor *Anopheles* biting activity stayed most likely unchanged during the rainy/transmission season from July to October ’96. Targeted studies may be needed to fully address this issue, however, we believe that our findings and the data recently reported on the IgG specific response to *An. gambiae* saliva in Angola [34] support the further development of arthropod salivary antigens as tools for the evaluation of the efficacy of vector control interventions by directly measuring its impact on the population and/or on single individuals.

Interestingly, the anti-gSG6 IgG response decreased with age, a pattern that is in contrast with the typical development of the humoral response to parasite antigens. Relatively poor information is currently available on the age distribution of the human humoral response to the *An. gambiae* saliva or salivary proteins. A progressive age-dependent decrease of the IgG response to *An. gambiae* saliva was previously reported, but this study was limited to Senegalese children 1 to 5 years old [10]. A similar gradual decline with age of IgE and IgG against *Aedes vexans* saliva was found in a group of Canadian children and blood donors aged from 1 month to 70 years [36], and this pattern was interpreted as natural desensitization to mosquito proteins. Human cutaneous reaction to mosquito bites, as originally described by Mellanby, undergoes five stages of skin reactivity [37], [38]: from initial no reaction of naïve hosts (stage I), to a delayed-type reaction (II) followed, as exposure continues, by an immediate- plus delayed-type reaction (III) and then an immediate response only (IV). Finally, after repeated long-term exposure, the host may develop desensitization and no skin reaction is observed anymore (stage V). Experimental desensitization to mosquito bites has been observed in a prospective study on a human subject exposed to bites of *Culex quinquefasciatus* and the decline in IgE and IgG responses appeared correlated to skin desensitization [36], [39]. The reduction of the anti-gSG6 IgG response in adults as compared to children was a surprising and interesting aspect of our study, which certainly deserves further investigation. At the moment we can only make some speculation suggesting that the age-related pattern of the anti-gSG6 IgG response found in our study may depend from the continued, intense exposure to bites of anopheline mosquitoes and may be explained in terms of development of immune tolerance to insect bite/saliva. However, the immune mechanisms underlying the development of natural desensitization to mosquito saliva are not well understood and future, targeted studies, will be necessary to clarify this aspect and get a deeper understanding of this phenomenon. In this perspective the *An. gambiae* gSG6 may represent an excellent model system to dissect molecular and cellular events leading to immune tolerance to insect saliva in humans.

In conclusion this is the first study where a mosquito recombinant salivary protein is used for a large scale analysis of the human humoral response in a malaria endemic area. The gSG6 protein proved to be a sensitive and reliable marker of human exposure to *An. gambiae*, suggesting it may be exploited for the development of risk maps and evaluation of vector control interventions, although experimental design should take into proper consideration age and, whenever possible, ethnicity. We analyzed here the humoral response to gSG6 at a micro-geographic scale in a condition of high levels of malaria transmission and vector exposure. It will be important to validate the gSG6 protein at a macro-geographic level and in conditions of low transmission/exposure; in this respect the identification and testing of additional salivary proteins with different immunogenic properties may prove very useful and, moreover, the use of combination(s) of antigens may help improving sensitivity. The availability of a repertoire of *An. gambiae* recombinant salivary proteins may also allow for their inclusion in protein microarrays carrying *Plasmodium* antigens, which are currently under development [40].

The *An. gambiae* gSG6 used in this study is 98% identical to the homolog from *Anopheles arabiensis* (GI: 225572575), a relevant malaria vector and member of the *An. gambiae* species complex [21]. Comparison to the *Anopheles funestus* protein (GI: 114864550) shows an identity of approximately 80% [17] and, therefore, it is reasonable to expect at least a partial cross-recognition by immune sera of the *An. gambiae* and the *An. funestus* proteins. These species are by far the most important African malaria vectors and, although this will need further experimental validation, it is likely that the *An. gambiae* gSG6 may represent a reliable marker of exposure to bites of the three main Afrotropical malaria vectors. While a gSG6 homolog is absent in the South American malaria vector *Anopheles darlingi* [15], the Asian mosquito *Anopheles stephensi* carries in its saliva a member of the gSG6 protein family [16] and, therefore, our observations may also be useful for the development of markers of exposure to Asian malaria vectors.

Finally, the age-related pattern of the anti-gSG6 humoral response reported here it is unusual and intriguing because the IgG response was very high in young children and progressively declined during adult age. The most likely explanation of this trend is the progressive desensitization to salivary proteins, a phenomenon that is known to occur after intense and prolonged exposure to insect bites, as is the case for the population studied here. The analysis of anti-gSG6 IgG subclasses, and eventually of IgE, may help better understanding this response pattern. Presently the knowledge concerning the development of immune tolerance to insect saliva in humans is still limited and, therefore, the gSG6 protein may also represent an attractive model system to get deeper insights into molecular and cellular mechanisms underlying this phenomenon.

## Materials and Methods

### Study Area, Subjects and Entomological Observations

Surveys were carried out in the rural villages of Barkoumbilen and Barkoundouba (∼35 km NE of Ouagadougou, Burkina Faso), inhabited by Mossi, Rimaibé and Fulani ethnic groups. As previously reported [24], [25] the area is characterized by intense *P. falciparum* transmission particularly during the June–October rainy season (entomological inoculation rates >100/person/year). Malaria prevalence is very high, *P. falciparum* representing about 95% of malaria infections. During the high transmission season, *P. falciparum* infection rates range (according to age group) from 60% to 90% in the Mossi-Rimaibé group, and from 20% to 80% amongst Fulani. Lower prevalences are observed during the dry low transmission season ranging between 40% and 80% amongst Mossi and Rimaibé and between 0% and 60% in Fulani. The study protocol was approved by the Technical Committee of the Centre National de Lutte contre le Paludisme of the Ministry of Health of Burkina Faso. Oral informed consent for multiple immuno-parasitological, clinical and entomological surveys was obtained from a Fulani community living in the village of Barkoundouba and from a Mossi community living 5 km apart in the village of Barkoumbilen. A total of 2066 sera collected during seven surveys in three consecutive years (August ’94, October ’94, March ’95, August ’95, October ’95, August ’96, October ’96) were analyzed in this study. Samples size and average age for each survey can be found in the legends to Figures. Sera from 60 Roman citizens (1–77 years old) who went to a city hospital for routine blood tests were used as a control. Entomological measures were based on indoor pyrethrum spray catches carried out monthly between August and November ‘94, in March ‘95, and between July and October ‘95 and ‘96. The main malaria vectors in the study area were *An. gambiae*, *An. arabiensis* and *An. funestus*; the latter represented <5% in most cases, reaching a maximum of ∼18% only in October-November ‘94. Permethrin-impregnated-curtains were installed in the two villages before the beginning of the high malaria transmission season, between the June 17 and 20, 1996. Additional details on study site and on entomological and parasitological aspects has been previously reported and can be found elsewhere [24]–[26].

### Protein Expression and Purification

The *An. gambiae* gSG6 was expressed as N-terminal His-tagged recombinant protein in the *E. coli* vector pET28b(+) (Novagen). Briefly, the region encoding the mature *An. gambiae* gSG6 protein was PCR amplified using the pPICZaA-G6 plasmid as template [21], a proofreading DNA polymerase (*Pfx*, Invitrogen) and the oligonucleotide primers G6F-Nde (5′-GTCTCATATGGAAAAGGTGTGGGTCGACCG-3′OH) and G6R-Eco (5′-GTCTGAATTCAATTACTGCTCCAGGAAGGCCTG-3′OH). Directional cloning in the *Nde*I/*Eco*RI-digested pET28b vector yielded the pET-gSG6 expression vector, that was sequenced and then introduced into competent BL21(DE3)RIL *E. coli* cells (Stratagene). After over night growth (37°C, LB medium) 5 ml of the saturated culture were transferred into 500 ml of LB and grown up to 0.8 OD_600_ before starting induction by IPTG (0.5 mM). After 3 hours cells were harvested and the pellet resuspended in 30 ml of 50 mM Tris-HCl pH 8.0, 50 mg/ml lysozyme and sonicated. Inclusion bodies (IB) were collected by centrifugation (13000 g, 20 min, 4°C), resuspended in extraction buffer (50 mM Tris-HCl pH 8.0, 2 M Urea, 5 mM EDTA, 1% Triton-X100), washed twice (50 mM Tris-HCl pH 8.0, 2 M Urea) and centrifuged as above. Proteins from IB were solubilized over-night (20 ml of 20 mM Na_2_HPO_4_, 6 M Guanidine-HCl, 0.5 M NaCl, 5 mM Imidazole, pH 7.4), centrifuged (20000 g, 30 min, 4°C) and subjected to affinity chromatography under denaturing conditions (HisTrap, GE Healthcare) according to manufacturer instructions. Fractions containing the His-tagged gSG6 were pooled, concentrated by Amicon® Ultra (Millipore, 10 kDa cut-off), refolded by dilution drop-by-drop in 20 volumes of refolding buffer (100 mM Tris-HCl pH 8.0, 500 mM L-Arg, 300 mM NaCl, 5 mM L-Glutathione reduced, 0.5 mM L-Glutathione oxidized) and left for an additional 24 to 48 h at 4°C with low stirring. After centrifugation (20000 g, 30 min, 4°C) the refolded proteins were concentrated by ultra-filtration (Ultracell 400, BioMax 10, Millipore), dialyzed against 20 mM Tris-HCl pH 7.5 and further purified by anion exchange chromatography (HiTrapQ, GE Healthcare). Elution was carried out with a linear gradient 0–0.5 M NaCl in 20 column volumes. Fractions containing the gSG6 recombinant protein were identified by SDS-PAGE, pooled, dyalized against 40 mM Tris-HCl pH 7.5 and quantified by the Bradford Protein Assay (Bio-Rad Laboratories). Homogeneity of the purified protein was assessed by silver staining and further confirmed by RP-HPLC and mass spectrometry (CEINGE Biotecnologie Avanzate, Naples, Italy). The typical yield of purified protein was of approximately 9–12 mg/l.

### Enzyme-Linked ImmunoSorbent Assay

ELISA was performed according to standard procedures. Maxisorp 96-well plates (Nunc M9410) were coated overnight (4°C, 10 µg/ml gSG6 in 15 mM Na_2_CO_3_, 35 mM NaHCO_3_, 3 mM NaN_3_, pH 9.6). After four washings wells were blocked (3 hrs, RT) in 150 µl of 1% w/v skimmed dry milk in PBST (PBS Sigma P4417+0.05% Tween 20), washed again and incubated overnight at 4°C with 50 µl of serum (1∶100) in blocking buffer. Serum samples were analyzed in duplicate with the antigen and once without antigen (coating buffer only). After washings plates were incubated (3 hrs, RT) with 100 µl of polyclonal rabbit anti-human IgG/HRP antibody (Dako P0214, 1∶5000 in blocking buffer). After washing as above the colorimetric development was carried out (15 min, RT in the dark) with 100 µl of *o*-phenylenediamine dihydrochloride (OPD, Sigma P8287). The reaction was terminated adding 25 µl of 2 M H_2_SO_4_ and the OD_492_ was determined using a microplate reader. IgG levels were expressed as final OD calculated for each serum as the mean OD value with antigen minus the OD value without antigen. Intra and inter assay variation of standard samples was below 20%. Sera whose duplicates showed a coefficient of variation (CV) ≥20% were not included in the analysis. The mean OD of unexposed controls plus 3 SD was used as cut-off value for seropositivity. A total of 2066 sera from exposed individuals and 60 from unexposed controls were analyzed by ELISA.

### Data Analysis

Anti-gSG6 IgG levels among two independent groups were compared by the Mann-Whitney U test. Multiple comparisons were performed by the Kruskal–Wallis test with the Dunn's post test correction for comparisons of pair of groups. The Wilcoxon matched-pairs test was used for comparison of two paired groups. Frequencies were compared by the chi-square test. All statistical analysis was performed using GraphPad Prism 5.0® statistical software (GraphPad Software Inc., La Jolla, CA).

## Supporting Information

Table S1 **Anti-gSG6 IgG response in children (1–10 years) and adults (>20 years).**
(DOC)

Text S1 **Supplementary info to** **Figure 6** **, panel B: number of individuals analyzed.**
(DOC)
