# Supplementary material for: Humoral Response to the Anopheles gambiae Salivary Protein gSG6: A Serological Indicator of Exposure to Afrotropical Malaria Vectors
Source: PLoS One. 2011 Mar 17;6(3):e17980. doi: 10.1371/journal.pone.0017980 (PMC3060095; doi:10.1371/journal.pone.0017980)
Supplement: Table S1 — Anti-gSG6 IgG response in children (1–10 years) and adults (>20 years). (DOC) [file pone.0017980.s001.doc]

Table S1. Anti-gSG6 IgG response in children (1-10 years) and adults (>20 years).

|  |  |  | **Median OD values** |  |  | **Prevalence** |  |
| --- | --- | --- | --- | --- | --- | --- | --- |
|  |  | **1-10 ^a^(n)^b^** | **>20 ^a^(n)^b^** | **p^c^** | **1-10 ^a^(n)^b^** | **>20 ^a^(n)^b^** | **p^d^** |
| Aug ‘94 | M | 0.614 (46) | 0.242 (27) | <0.0001 | 0.56 (82) | 0.36 (75) | 0.0181 |
|  | F | 0.714 (44) | 0.298 (18) | 0.0013 | 0.81 (54) | 0.47 (38) | 0.0013 |
| Oct ‘94 | M | 0.535 (52) | 0.327 (20) | 0.0265 | 0.69 (75) | 0.62 (32) | ns |
|  | F | 0.686 (18) | 0.342 (11) | ns | 0.90 (20) | 0.52 (21) | 0.0212 |
| Mar ‘95 | M | 0.290 (20) | 0.229 (15) | ns | 0.46 (43) | 0.47 (32) | ns |
|  | F | 0.661 (27) | 0.414 (4) | ns | 0.90 (30) | 0.23 (17) | <0.0001 |
| Aug ‘95 | M | 0.592 (75) | 0.346 (38) | 0.0055 | 0.66 (113) | 0.47 (80) | 0.0133 |
|  | F | 0.646 (53) | 0.366 (14) | 0.0291 | 0.90 (59) | 0.64 (22) | 0.0167 |
| Oct ‘95 | M | 0.519 (77) | 0.328 (26) | 0.0234 | 0.79 (98) | 0.67 (39) | ns |
|  | F | 0.925 (45) | 0.378 (33) | <0.0001 | 0.88 (51) | 0.61 (54) | 0.0031 |
| Aug ‘96 | M | 0.414 (23) | 0.405 (10) | ns | 0.72 (32) | 0.37 (27) | 0.0154 |
|  | F | 0.958 (20) | 0.379 (7) | ns | 1.00 (20) | 0.41 (17) | <0.0001 |
| Oct ‘96 | M | 0.505 (21) | 0.342 (9) | ns | 0.49 (43) | 0.31 (29) | ns |
|  | F | 0.718 (55) | 0.633 (21) | 0.0470 | 0.93 (59) | 0.44 (48) | <0.0001 |

M, Mossi; F, Fulani.

(a) Years of age.

(b) n=number of individuals.

(c) Mann-Withney.

(d) Yates corrected chi-square.
