# Supplementary material for: Humoral Response to the Anopheles gambiae Salivary Protein gSG6: A Serological Indicator of Exposure to Afrotropical Malaria Vectors
Source: PLoS One. 2011 Mar 17;6(3):e17980. doi: 10.1371/journal.pone.0017980 (PMC3060095; doi:10.1371/journal.pone.0017980)
Supplement: Text S1 — Supplementary info to Figure 6 , panel B: number of individuals analyzed. (DOC) [file pone.0017980.s002.doc]

**Text S1. Supplementary info to Figure 6, panel B: number of individuals analyzed.**

The number of individuals analyzed for each survey were as follows:

**August 94’** (**Mossi**: 1-5, n=38; 5-10, n=44; 10-20, n=48; 20-40, n=46; >40, n=29. **Fulani**: 1-5, n=22; 5-10, n=32; 10-20, n=39; 20-40, n=26; >40, n=12);

**October ’94** (**Mossi**: 1-5, n=39; 5-10, n=36; 10-20, n=27; 20-40, n=22; >40, n=10. **Fulani**: 1-5, n=8; 5-10, n=12; 10-20, n=16; 20-40, n=14; >40, n=7);

**March ’95** (**Mossi**: 1-5, n=25; 5-10, n=18; 10-20, n=23; 20-40, n=21; >40, n=11. **Fulani**: 1-5, n=12; 5-10, n=18; 10-20, n=18; 20-40, n=12; >40, n=5);

**August ’95** (**Mossi**: 1-5, n=62; 5-10, n=51; 10-20, n=60; 20-40, n=49; >40, n=31. **Fulani**: 1-5, n=21; 5-10, n=38; 10-20, n=36; 20-40, n=21; >40, n=1);

**October ’95** (**Mossi**: 1-5, n=47; 5-10, n=51; 10-20, n=38; 20-40, n=27; >40, n=12. **Fulani**: 1-5, n=20; 5-10, n=31; 10-20, n=36; 20-40, n=36; >40, n=18);

**August ’96** (**Mossi**: 1-5, n=13; 5-10, n=19; 10-20, n=13; 20-40, n=17; >40, n=10. **Fulani**: 1-5, n=9; 5-10, n=11; 10-20, n=20; 20-40, n=11; >40, n=6);

**October ’96** (**Mossi**: 1-5, n=8; 5-10, n=35; 10-20, n=33; 20-40, n=15; >40, n=14. **Fulani**: 1-5, n=16; 5-10, n=43; 10-20, n=35; 20-40, n=27; >40, n=21).
